# The Golgin Protein Giantin Regulates Interconnections Between Golgi Stacks

**DOI:** 10.3389/fcell.2019.00160

**Published:** 2019-08-27

**Authors:** Ayano Satoh, Mitsuko Hayashi-Nishino, Takuto Shakuno, Junko Masuda, Mayuko Koreishi, Runa Murakami, Yoshimasa Nakamura, Toshiyuki Nakamura, Naomi Abe-Kanoh, Yasuko Honjo, Joerg Malsam, Sidney Yu, Kunihiko Nishino

**Affiliations:** ^1^Graduate School of Interdisciplinary Science and Engineering in Health Systems, Okayama University, Okayama, Japan; ^2^Institute of Scientific and Industrial Research, Osaka University, Osaka, Japan; ^3^Graduate School of Natural Science and Technology, Okayama University, Okayama, Japan; ^4^Graduate School of Environmental and Life Science, Okayama University, Okayama, Japan; ^5^Department of Public Health and Applied Nutrition, Institute of Biomedical Sciences, Graduate School Tokushima University, Tokushima, Japan; ^6^Research Institute for Radiation Biology and Medicine, Hiroshima University, Hiroshima, Japan; ^7^Center for Biochemistry (BZH), Heidelberg University, Heidelberg, Germany; ^8^School of Biomedical Sciences, The Chinese University of Hong Kong, Hong Kong, Hong Kong

**Keywords:** Golgi, golgins, glycosylation, endoplasmic reticulum, electron tomography

## Abstract

Golgins are a family of Golgi-localized long coiled-coil proteins. The major golgin function is thought to be the tethering of vesicles, membranes, and cytoskeletal elements to the Golgi. We previously showed that knockdown of one of the longest golgins, Giantin, altered the glycosylation patterns of cell surfaces and the kinetics of cargo transport, suggesting that Giantin maintains correct glycosylation through slowing down transport within the Golgi. Giantin knockdown also altered the sizes and numbers of mini Golgi stacks generated by microtubule de-polymerization, suggesting that it maintains the independence of individual Golgi stacks. Therefore, it is presumed that Golgi stacks lose their independence following Giantin knockdown, allowing easier and possibly increased transport among stacks and abnormal glycosylation. To gain structural insights into the independence of Golgi stacks, we herein performed electron tomography and 3D modeling of Golgi stacks in Giantin knockdown cells. Compared with control cells, Giantin-knockdown cells had fewer and smaller fenestrae within each cisterna. This was supported by data showing that the diffusion rate of Golgi membrane proteins is faster in Giantin-knockdown Golgi, indicating that Giantin knockdown structurally and functionally increases connectivity among Golgi cisternae and stacks. This increased connectivity suggests that contrary to the *cis*-golgin tether model, Giantin instead inhibits the tether and fusion of nearby Golgi cisternae and stacks, resulting in transport difficulties between stacks that may enable the correct glycosylation of proteins and lipids passing through the Golgi.

## Introduction

Eukaryotic cells have various forms of glycans on their cell surfaces that are important for cell–cell communications, development, differentiation, infection, and signaling. Most of these glycans are attached to lipids and proteins initially in the endoplasmic reticulum (ER) and are further extended and trimmed in the Golgi apparatus.

Glycan structures vary depending on cell types and species. One of the reasons for such structural variation is differences in the expression patterns of glycosyltransferases and glycosidases responsible for glycan biosynthesis, although it may also depend on the structure of the Golgi (Koreishi et al., 2013a; Xiang et al., 2013). The Golgi apparatus is usually a pancake-like structure consisting of a stack of several flat membrane cisternae, which is further linked laterally and forms a ribbon-like structure [Golgi ribbon (Wei and Seemann, 2010; Gosavi and Gleeson, 2017)]. The stacking of Golgi cisternae is secured by Golgi stacking proteins GRASP55/65, and their loss disrupts Golgi stacking and accelerates cargo transport without affecting the lateral linking (ribbon formation) of the stacks (Xiang et al., 2013). Glycan analyses showed that the loss of GRASP55/65 decreased the cell surface expression of high-mannose- and complex-type glycans, which are formed in the Golgi (Xiang et al., 2013). The authors of this study proposed that GRASP55/65 may slow cargo transport down to ensure correct glycosylation occurs through the Golgi by maintaining Golgi stacks in normal cells (Xiang et al., 2013).

Similar to this study, we previously reported that loss of Giantin, the longest golgin which is a family of Golgi-localized coiled-coil proteins, also accelerates cargo transport by affecting lateral linking of the stacks (Koreishi et al., 2013a). Lectin staining showed that the loss of Giantin increased the proportion of highly branched glycans with sialic acids, representing complex-type glycans (Koreishi et al., 2013a). The key difference between our finding and that of Xiang et al. with regard to glycan structures is whether lateral stack linking is also affected.

Giantin, also known as GCP364, was originally identified as a C-terminally anchored Golgi membrane protein (Linstedt and Hauri, 1993; Linstedt et al., 1995; Toki et al., 1997). It is also recognized as an autoantigen in autoimmune diseases (reviews Stinton et al., 2004; Saraste, 2016). Golgins share predicted coiled-coil structures known to form long rod-like structures, which function in tethering vesicles, membranes, and other cytoskeletal factors. Giantin is thought to tether coatomer protein I (COPI)-coated vesicles to *cis*-Golgi membranes (Sönnichsen et al., 1998; Lesa et al., 2000; Linstedt et al., 2000; Shorter and Warren, 2002, the *cis*-golgin tether model). In this model, Giantin on COPI vesicles and golgin GM130 in *cis*-Golgi membranes are linked by a soluble protein, p115; therefore, COPI vesicles are tethered to *cis*-Golgi. Although Giantin localizes to both the Golgi and COPI vesicles, it is distributed asymmetrically, with more seen in COPI vesicles (Sönnichsen et al., 1998).

To understand the *cis*-golgin tether model, we previously performed RNA interference (RNAi) of Giantin. Without the tether, it was presumed that cells would be filled with untethered vesicles that may cause incorrect vesicle transport. Anterograde transport was accelerated and sialic acid-bearing cell surface glycans were increased by the loss of Giantin. However, we observed surprisingly few vesicles, which were likely untethered vesicles. This suggested that Giantin has other functions than the COPI vesicle tether. Indeed, we detected an alteration of lateral linking of the stacks following the loss of Giantin (Koreishi et al., 2013a). To gain insights into the structural alteration, which may have caused transport alteration, we herein performed 3D modeling of Golgi cisternae and stacks, and found them to be tightly linked laterally by Giantin depletion. This finding is supported by the observed increased lateral diffusion of Golgi membrane proteins.

## Materials and Methods

### Cell Lines, Culture, Small Interfering (si)RNA Transfection, and Fluorescence Recovery After Photobleaching (FRAP) Experiments

HeLa cells (CCL-2, ATCC, Manassas, VA) were maintained and transfected with Giantin siRNA as described previously (Koreishi et al., 2013a). HeLa cells stably expressing murine Golgi mannosidase II (ManII)-GFP (a gift from J. White, European Molecular Biology Laboratory, Heidelberg, Germany) were established as previously described (Maday et al., 2008; Koreishi et al., 2013b). FRAP experiments were performed as described in earlier studies (Rutz et al., 2009; Koreishi et al., 2013b). The relative diffusion rate and % maximum recovery were obtained by fitting the FRAP data to Ellenberg’s diffusion equation (Ellenberg et al., 1997) shown as below.

%FRAP = (%maximum recovery) ^∗^ (1−√((diffusion rate)/((diffusion rate) + π ^∗^ (time after recovery))))

### Conventional Electron Microscopy

Conventional electron microscopy was performed as previously described (Hayashi-Nishino et al., 2009) with slight modifications. In brief, cells were fixed in 2.5% glutaraldehyde in 0.1 M sodium phosphate buffer, pH 7.4 (PB) for 1 h. Cells were washed in PB, then scraped and collected as pellets. These were post-fixed in buffer containing 1% OsO_4_ and 0.5% potassium ferrocyanide, dehydrated in a series of graded ethanol solutions, followed by propylene oxide, and embedded in epoxy resin.

### Electron Tomography

Cell specimens prepared as above were cut into 300-nm thick sections, collected on formvar/carbon-coated grids, stained with uranyl acetate and lead citrate, and examined using a JEM-2100 transmission electron microscope (The Japan Electron Optics Laboratory Co., Ltd. (JEOL; Tokyo, Japan) at accelerating voltages of 200 kV. Tilt series data for each section were recorded around two orthogonal axes (1° interval over ± 60° for each axis) using a 2k × 2k CCD camera (Gatan US1000, Gatan Inc., Warrendale, PA). Tomograms were computed and joined to form a dual-axis tomogram using the IMOD software package. Subcellular structures within the 3D volume were segmented, and their surfaces were modeled with IMOD (Kremer et al., 1996; Mastronarde, 1997). Contours of membranes of the Golgi apparatus and other membrane structures visible at the Golgi regions were traced manually to generate the final models. Vesicles were represented by spheres.

### Cell Cycle Analysis

Cell cycle analysis was performed as described (Liu et al., 2017). Briefly, HeLa cells with or without Giantin RNAi were treated with trypsin-EDTA and fixed with ice-cold 70% ethanol overnight at –20°C. After washing with phosphate-buffered saline, cells were stained with 0.1 mg/ml propidium iodide (Thermo Fisher Scientific, Waltham, MA, United States) containing 0.1 mg/ml RNase A for 30 min. DNA contents in stained cells were measured by a Tali image-based cytometer (Thermo Fisher Scientific) or a BD Accuri C6 flow cytometer (Becton, Dickinson and Company, Franklin Lakes, NJ, United States).

### RNA Isolation and Quantitative (q)PCR

Total RNA was isolated using the Total RNA Extraction Kit (Viogene, New Taipei City, Taiwan) and reverse-transcribed using the SuperScript VILO cDNA Synthesis Kit (Thermo Fisher Scientific). Alternatively, total RNA extraction and reverse transcription were performed using the SuperPrep^®^ Cell Lysis & RT Kit for qPCR (Toyobo, Tokyo, Japan). The relative expression of mRNAs was quantified using the LightCycler^®^ 480 with SYBR Green I Master (Indianapolis, IN). Primer sets used were as follows: human beta-actin fwd: 5′-CCAACCGCGAGAAGATGA-3′ and human beta-actin rev: 5′-TCCATCACGATGCCAGTG-3′; human GALNT5 fwd: 5′-AGAGCCATTGAAGACACCAGA-3′ and human GALNT5rev: 5′-CACTGGTGGTTGGGAGGTTA-3′; human GALNT5 fwd2: 5′-CCAGTGGATAGAGCCATTGAA-3′ and human GALNT5 rev2: 5′-TGGTTGGGAGGTTATTGTGAA-3′; human ST6GALNAC3 fwd: 5′-CACAGAGAAGCGCATGAGTTA-3′ and human STGALNAC3 rev: 5′-TCTTCAAGGCGTGAACA AAA-3′; human EXTL1 fwd: 5′-TGTGAGCAAGACCCTGGAC-3′ and human EXTL1 rev: 5′-CCAGAGATGAGGCAGAAGGT-3′; and human ST6GAL2 fwd: 5′-TCATCCTAAATTTATA TGGCAGCTC-3′ and human ST6GAL2 rev: 5′-TGAGGA TGCCCAAGCAGT-3′.

### Statistical Analysis

All statistical comparisons in this study were performed using the Student’s *t*-test for independent samples or a non-parametric test, Mann-Whitney U Test calculated by an online calculator: https://www.socscistatistics.com/tests/mannwhitney.

## Results

### Loss of Giantin Elongates Golgi Cisternae

**FIGURE 1:**
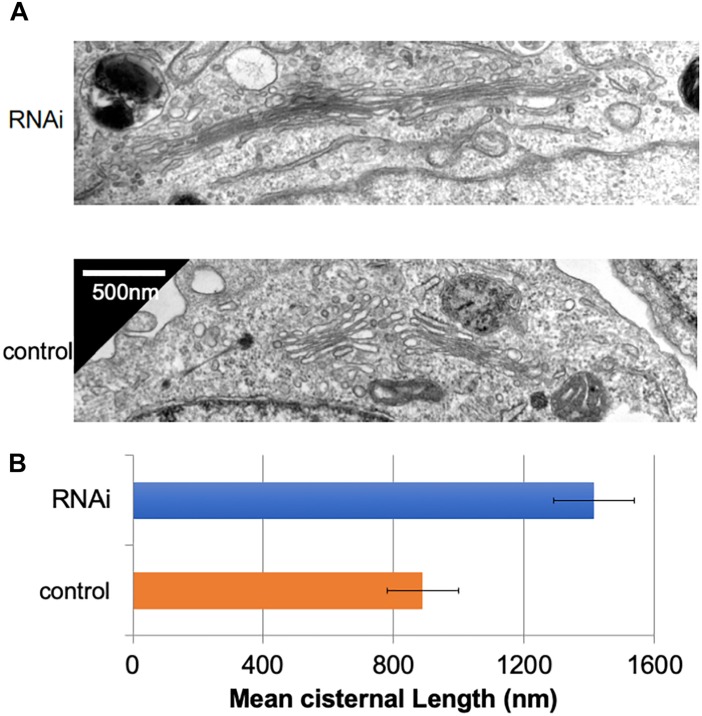
Loss of Giantin elongates Golgi cisternae. **(A)** Typical electron micrographs of Golgi cisternae in Giantin and control siRNA-treated cells. Bar, 500 nm. **(B)** Cisternal lengths were measured by tracing the cisternae manually using ImageJ software (**B**, *n* = 10 micrographs, bar, SEM, *P* < 0.00001).

In many mammalian cells, Golgi stacks are connected as ribbon-like structures that are dependent on the integrity of microtubules, the depolymerization of which by Nocodazole disperses the ribbon-like Golgi into mini Golgi stacks that can be observed by light microscopy. Our previous work using Nocodazole suggested that the connectivity between Golgi stacks may be altered by the loss of Giantin (Koreishi et al., 2013a). To gain insights into these changes associated with the loss of Giantin, we performed conventional electron microscopy of Giantin siRNA-treated and control cells (Figure 1A). All the efficiencies of Giantin knockdown shown in this study were >90% as estimated by immunofluorescence (Supplementary Figure S1). Compared with control cells, Giantin siRNA-treated cells had Golgi cisternae that were around 1.5 times longer (Figure 1B). Supplementary Figure S2 depicts the distribution of the cisternal lengths as a histogram. Because there was no significant change in the numbers of Golgi cisternae per stack following Giantin RNAi (Supplementary Figure S3), the observed elongation is likely the result of lateral fusions of Golgi cisternae rather than increased membranes in Golgi areas. Importantly, other siRNA to Giantin (siRNA5) also had the similar effect on cisternal lengths, and exogenous expression of rat Giantin, which is resistant to siRNAs used, reduced the effect partly (Supplementary Figure S4).

### Loss of Giantin Connects or Fuses Golgi Cisternae and Stacks

**FIGURE 2:**
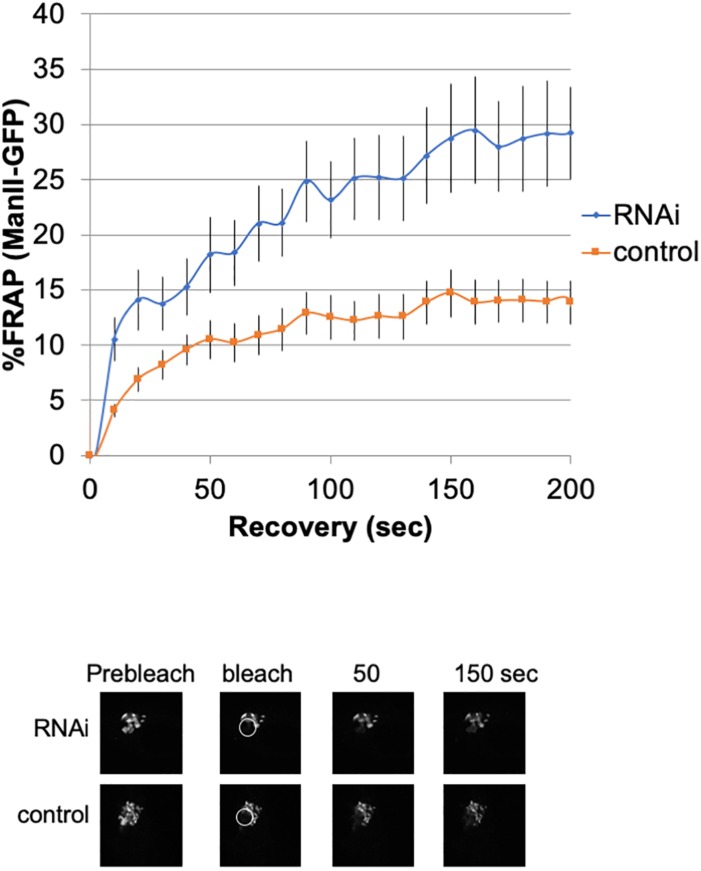
Loss of Giantin changes the intra-connectivity between Golgi cisternae/stacks. HeLa cells stably expressing ManII-GFP with Giantin or control siRNA treatment were subjected to FRAP experiments. GFP-labeled Golgi in ManII-GFP expressing cells was photobleached, and fluorescence recoveries were monitored for 200 s and graphed (*n* = 10, bar, SD). Representative images of fluorescence recovery at the indicated time points are shown below. Sizes 25.5 (w) × 25.5 (h) μm. The circles represent the bleached area.

To investigate the lateral fusion of Golgi cisternae in living cells, we performed FRAP of the Golgi membrane protein ManII-GFP (Storrie et al., 1998; Ward et al., 2001). As shown in Figure 2, FRAP of ManII-GFP in Giantin siRNA-treated cells was much faster than that in control cells. The FRAP data were curve-fitted to Ellenberg’s diffusion equation revealing that the relative diffusion rate of ManII-GFP in Giantin-siRNA-treated Golgi was 2.3-fold compared to the control. These data suggested that Golgi proteins in Giantin siRNA-treated Golgi move faster and more readily than in control cells, indicating that the connectivity between Golgi cisternae and stacks was functionally increased by the loss of Giantin. Importantly, other siRNA to Giantin (siRNA5) also had the similar effect on FRAP, and exogenous expression of rat Giantin, which is resistant to siRNAs used, reduced the effect partly (Supplementary Table S1).

**FIGURE 3:**
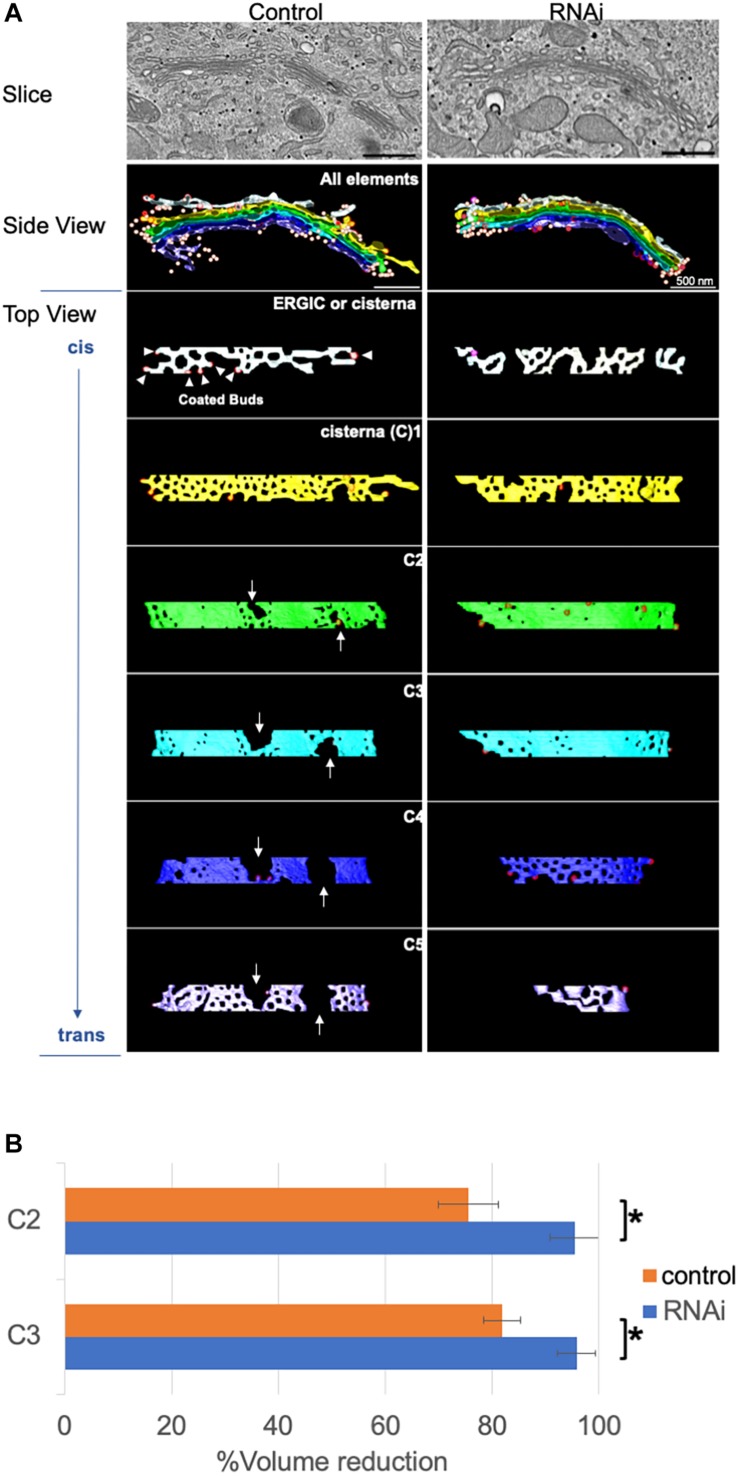
Loss of Giantin fuses Golgi cisternae. **(A)** Typical 3D models of Golgi cisternae in Giantin and control siRNA-treated cells. One of the tomographic slices used for these 3D models is shown in top panels. Note that the Golgi cisternae visible in RNAi cells appear longer than those in control cells. Bar, 500 nm. Arrows indicate large fenestrae in cisternae. The *cis*-most and *trans*-most cisternae are labeled C1 and C5, respectively. **(B)** The reduction of cisternal volumes from the corresponding no-fenestra model is shown as the % volume reduction in Giantin and control siRNA-treated cells. Bar, SD (*n* = 4, ^∗^*P* < 0.005).

For further structural analysis, we carried out 3D modeling of electron tomograms of Golgi cisternae/stacks following the loss of Giantin. Approximately 10 tomograms were used for 3D modeling by IMOD software for 3D reconstruction of EM serial sections. Compared with Golgi cisternae of control cells, those of Giantin siRNA-treated cells were much smoother with fewer and smaller wells [Rambourg and Clermont (1990) and fenestrae which were previously defined as “non-compact regions” (Ladinsky et al., 1999; Figure 3A)]. Of note, one of the tomographic slices used in 3D modeling shown in the top panels of Figure 3A reveals longer cisternae in Giantin siRNA-treated cells than those in control cells. This is in a good agreement with Figure 1A. Examples of tomograms and modeling are shown in Supplementary Movies and Supplementary Figure S5.

The smoothness of the cisternae was then quantified by comparing the volumes of 3D models without fenestrae to with fenestrae (Figure 3B). In this quantification, the difference between cisternal volumes of the actual model and those of the no-fenestra model indicates the volumes of fenestrae. An example of the no-fenestra model is shown in Supplementary Figure S6. The volume differences, i.e., the volume of the fenestrae, in control cells depended on the positions of the cisternae (C2–C3), but were approximately 20∼30%. In contrast, the differences in Giantin siRNA-treated cells were less than 10%, which is indicative of smoother cisternae. We found no significant difference in C1 and C4 cisternae because of their heterogeneity. We also tried to quantify the smoothness by comparing surface areas to volumes. If the structure has a rough surface, the ratio between the surface area and volume would be higher than if the structure was smooth. However, because cisternae appeared to be thicker near the fenestrae (Supplementary Figure S7), we concluded that quantification of smoothness in this way is not appropriate (data not shown). The total volumes (including those of un-modeled fenestrae) of C2 cisternae are 0.011 ± 0.006 μm^3^ and 0.0097 ± 0.003 μm^3^ for control and RNAi cells, respectively. Importantly, the loss of Giantin did not change cell cycle progression (data not shown).

## Discussion

Our previous data suggested that the Golgi stack loses its independence of individual Golgi stacks following Giantin knockdown, allowing easier transport among Golgi stacks that may be responsible for increased transport and abnormal glycosylation (Koreishi et al., 2013a). In this study, we confirmed by 3D modeling of electron tomograms that Golgi stacks and cisternae have less independence after the loss of Giantin.

Giantin has a proposed function as a *cis*-Golgin tether which tethers COPI vesicles to *cis*-Golgi membranes (Sönnichsen et al., 1998; Linstedt et al., 2000; Alvarez et al., 2001). In the present study, we modeled vesicles around Golgi stacks but were unable to see more untethered COPI vesicles after the loss of Giantin (Supplementary Figure S8). To test whether the loss of Giantin increases untethered COPI vesicles, we may need to use larger specimens for future modeling.

### Golgi 3D Structures

3D Golgi modeling has previously revealed novel Golgi structures including connected Golgi cisternae, branched Golgi cisternae, and autophagosome-like Golgi (reviews Mogelsvang et al., 2004; Marsh, 2005; Martínez-Alonso et al., 2013). Among these works, the first and most crucial examination was 3D modeling of the Golgi in normal rat kidney cells (Ladinsky et al., 1999). Importantly, this work quantitatively described non-compact regions that include unconnected cisternae, some vesicles, and fenestrae. We also identified this “non-compact region” between Golgi stacks in HeLa cells in the present study, although few vesicles were observed in this region.

Our comparison of cisternal volumes between actual and no-fenestra models revealed a relative volume of the non-compact region in control HeLa cells of around 20% (Figure 3). This volume was reduced by the loss of Giantin, suggesting that Giantin plays an essential role in the generation of the non-compact region although the function of this region remains unclear. Extrapolating from our previous work showing that the loss of Giantin increases cargo transport and affects cell surface glycans, we speculate that the non-compact region organizes transport through the Golgi. Further, our previous work by 2D immuno-EM showed that Giantin localizes to the rims of Golgi cisternae that may be part of the fenestrae/non-compact regions. It is noteworthy that depletion of the Golgi-localized small GTPase Rab6 increased the lateral continuity of the Golgi (Storrie et al., 2012), and Rab6 was also shown to interact with Giantin (Rosing et al., 2007). Taken together, Giantin may function together with Rab6 in the maintenance of the non-compact region. Another possibility is that the loss of Giantin reduces fission of the Golgi cisternae or changes in the flux of vesicle transport within the Golgi. Although we are unable to test this possibility, the loss of Giantin did not increase the number of vesicles in the areas observed (Supplementary Figure S8), suggesting that these may be comparable with or without Giantin.

Our previous work showed that the loss of Giantin altered lateral connectivity of Golgi stacks using microtubule depolymerizer, Nocodazole. Although this appeared to contradict the present study, the previous study, in particular, the EM study was done in the presence of Nocodazole suggesting that microtubules may contribute to the Giantin-regulated lateral connectivity. From this viewpoint, the Golgi-microtubule associate proteins (GMAPs) together with Giantin may also play a key role in the independence of Golgi cisternae, but we have not identified such GMAPs, yet. Our updated working model is shown in Supplementary Figure S10.

### Giantin and Glycosylation

Our previous data showed that the loss of Giantin altered the glycosylation patterns of cell surface proteins (Koreishi et al., 2013a). Golgins, including Giantin, have been shown to interact with some glycosyltransferases and are required for their targeting to functional sites (Petrosyan et al., 2012). These golgin-interacting glycosyltransferases are responsible for the biosynthesis of mucin-type glycoproteins. We did not test whether the localization of these enzymes or the surface expression of mucin-type glycoproteins is changed after the loss of Giantin, but the observed alterations in glycosylation patterns might reflect the targeting failure of glycosyltransferases.

Another study showed that loss of Giantin by clustered regularly interspaced short palindromic repeats (CRISPR)/CRISPR-associated protein 9 affected glycosyltransferase expression levels in the RPE-1 cell line derived from human retina (Stevenson et al., 2017). We also tested those glycosyltransferase expression levels in HeLa cells after Giantin RNAi, but found no significant changes (Supplementary Figure S9). The authors also claimed that the knock-out of Giantin by CRISPR in RPE1 cells did not change the cisternal lengths and cargo transport. Therefore, the phenotypes in cisternal lengths, glycosylation, and transport that we observed by the knock-down of Giantin with siRNAs can be specific to HeLa cells.

### Cell-Type Specific Functions of Giantin

Giantin is expressed ubiquitously (Fagerberg et al., 2014), and is widely used as a Golgi marker protein. Giantin knock-out or mutant animal models have revealed the cell-type specific function of Giantin (Katayama et al., 2011, 2018; Lan et al., 2016; Bergen et al., 2017; McGee et al., 2017; Stevenson et al., 2017). Moreover, Giantin gene mutations and pathogenic changes in Giantin levels have been reported in various diseases, including sickle cell disease (Alsultan et al., 2018; Amlie-Lefond et al., 2018), bipolar disorder (Kerner et al., 2013), hepatocellular carcinoma (Choi et al., 2017), leukemia (Troadec et al., 2017), pulmonary disease (Raparelli et al., 2018), and post-alcohol recovery (Casey et al., 2018). These various phenotypes might be caused by the expression of Giantin binding partners, including GCP60 (Sohda et al., 2001), RCAN2 (Stevenson et al., 2018), PRMT5 (Sohail et al., 2015), PLK3 (Ruan et al., 2004), Rab6, p115, and Rab1 (Beard et al., 2005). In the present study, we noticed that immunofluorescence of GCP60 around the Golgi was less distinct after the loss of Giantin (data not shown). Therefore, GCP60 may function together with Giantin in the independence of Golgi stacks.

## Conclusion and Perspectives

Our study found that Golgi stacks and cisternae changed their structure following the loss of Giantin, which may correlate with our previous finding of the alteration of glycosylation patterns of cell surface proteins. This is reasonable considering that glycosylation is a series of chemical reactions and that Golgi stacks and cisternae are reaction vessels. In other words, structural changes to reaction vessels by the lateral linking of Golgi cisternae may alter temporal and local concentrations of enzymes and substrates for glycosylation reactions, which may result in changes to surface glycosylation patterns and secretion.

The correlation between structural changes of the Golgi and glycosylation patterns was previously explained as a result of ‘sorting zones’ in the Golgi (Yano et al., 2005) in which each zone has a unique role in providing glycosylation and sorting. For example, one zone is responsible for making glycosaminoglycans, and the others are responsible for protein *N*-glycosylation or GPI-anchoring. It could be speculated that these sorting zones are lost following Giantin knockdown, leading to changes in surface glycosylation patterns. Future studies using recent technologies including super-resolution microscopy (Tie et al., 2018), cryo-focused ion beam scanning electron microscopy (FIB-SEM) with membrane segmentation by deep learning, and other new methods (Ranftler et al., 2017; Gorelick et al., 2019) will help dissect the structural changes associated with Golgi stacks and cisternae and sorting zones.

## Data Availability

The raw data supporting the conclusions of this manuscript will be made available by the authors, without undue reservation, to any qualified researcher.

## Author Contributions

MH-N, JoM, and AS: electron microscopy. TS, MH-N, KN, and AS: electron tomography and 3D modeling. MK, RM, YH, SY, and AS: giantin RNAi. YN, TN, NA-K, JuM, and AS: cell cycle analysis. JuM and AS: plasmid construction.

## Conflict of Interest Statement

The authors declare that the research was conducted in the absence of any commercial or financial relationships that could be construed as a potential conflict of interest.
