# Supplementary figures and images for: The Golgin Protein Giantin Regulates Interconnections Between Golgi Stacks
Source: Front Cell Dev Biol. 2019 Aug 27;7:160. doi: 10.3389/fcell.2019.00160 (PMC6732663; doi:10.3389/fcell.2019.00160)

**Supplementary Figures**

A (for Figure 1)

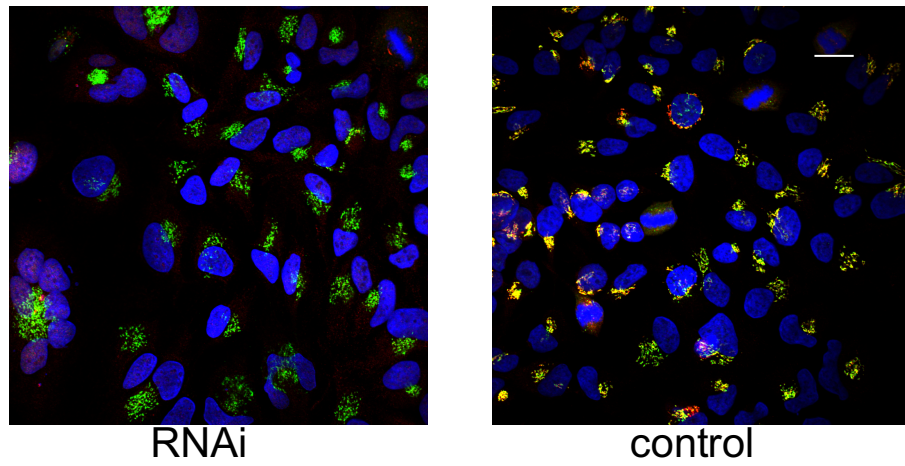

B (for Figure 2)

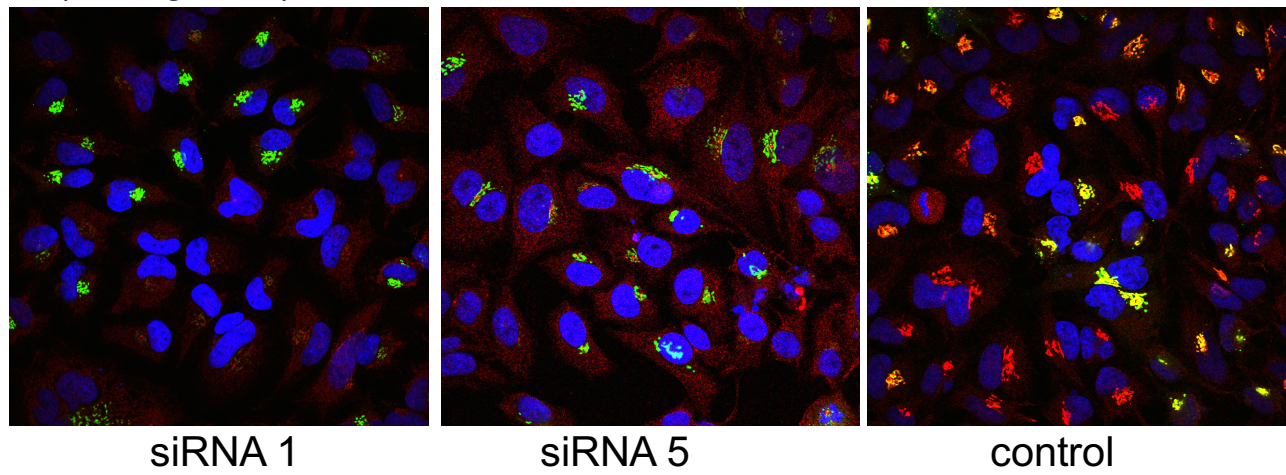

Supplement: Supplementary file 16 [file Data_Sheet_1.pdf]
