# Supplementary material for: The Golgin Protein Giantin Regulates Interconnections Between Golgi Stacks
Source: Front Cell Dev Biol. 2019 Aug 27;7:160. doi: 10.3389/fcell.2019.00160 (PMC6732663; doi:10.3389/fcell.2019.00160)
Supplement: Supplementary file 17 [file Data_Sheet_2.pdf]

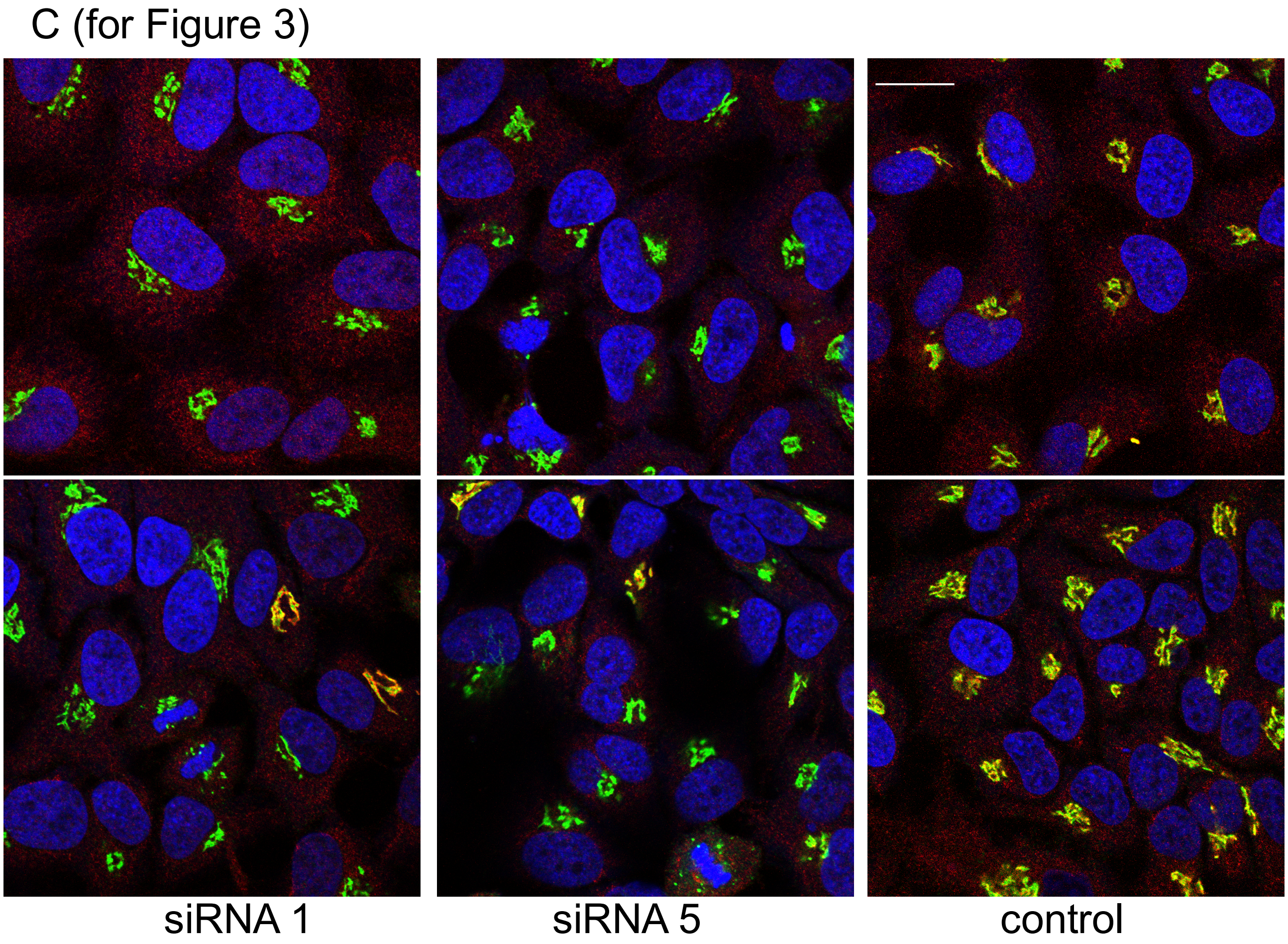

Supplementary Figure 1. Immunofluorescence to show knockdown efficiencies.

(A) Giantin and control siRNA-treated cells used for Figure 1 were subjected to indirect immunofluorescence for Giantin (red), GM130 (green), and nuclei (blue). Bar, 20 µm. (B) Giantin (siRNA1 and siRNA5) and control siRNA-treated cells used for Figure 2 were subjected to indirect immunofluorescence for Giantin (red), ManII-GFP (green), and nuclei (blue) shown with the same scale to (A). (C) Giantin (siRNA1 and siRNA5) and control siRNA-treated cells used for Figure 3 were subjected to indirect immunofluorescence for Giantin (red), GM130 (green), and nuclei (blue). Bar, 20 µm. Both upper and bottom panels are from the same coverslips but under different fields. Giantin-positive cells were rarely observed on the same coverslips, but some are shown in the bottom panels.
