# Supplementary material for: The Golgin Protein Giantin Regulates Interconnections Between Golgi Stacks
Source: Front Cell Dev Biol. 2019 Aug 27;7:160. doi: 10.3389/fcell.2019.00160 (PMC6732663; doi:10.3389/fcell.2019.00160)
Supplement: Supplementary file 18 [file Data_Sheet_3.pdf]

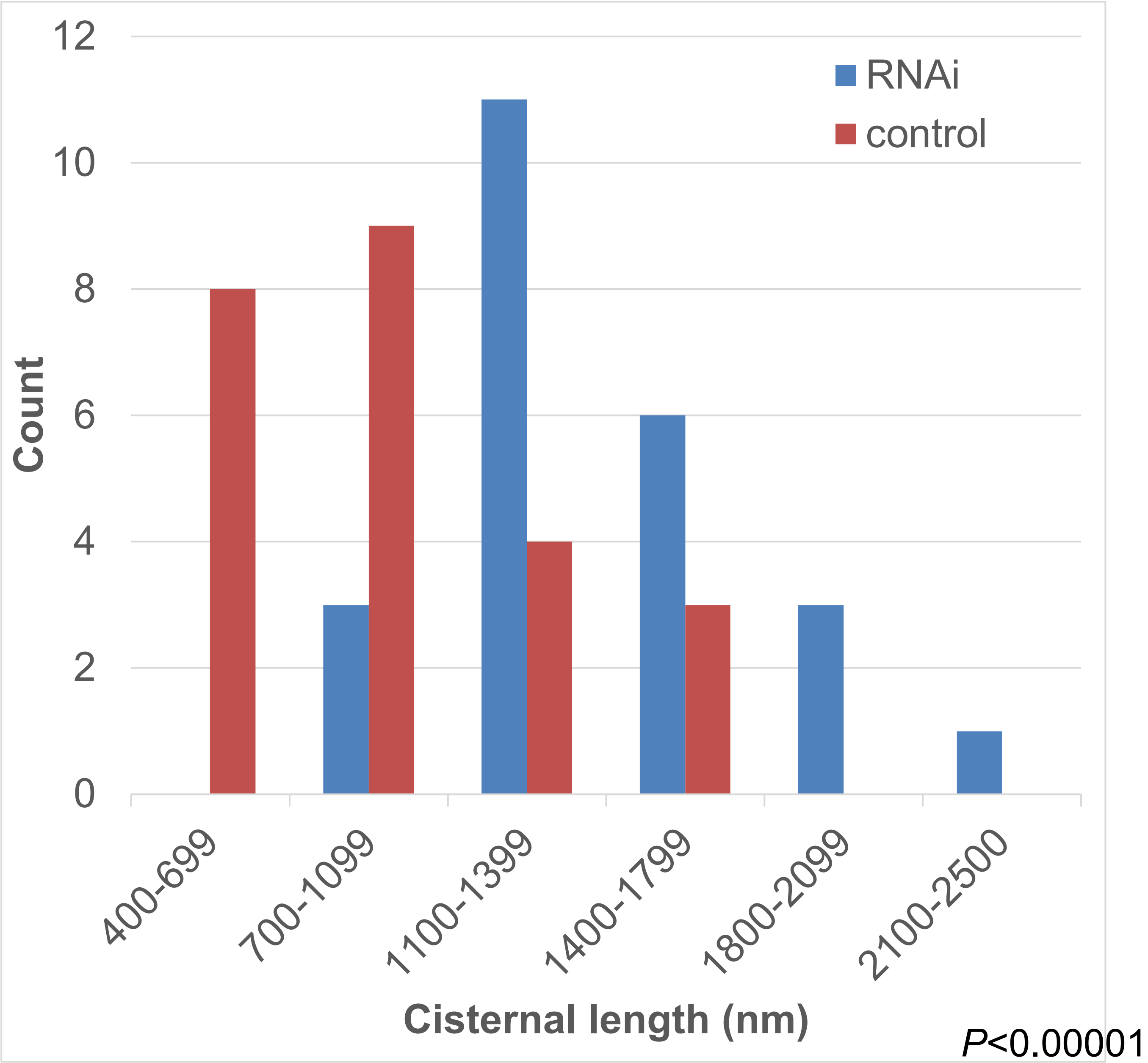

Supplementary Figure 2. Loss of Giantin elongates Golgi cisternae.

Distribution of the cisternal lengths shown in Figure 1. The histogram represents the distributions of cisternal length data shown in Figure 1 (n=24 from ~10 electron micrographs). $P<0.00001$ was calculated using an online calculator (https://www.socscistatistics.com/tests/mannwhitney/).

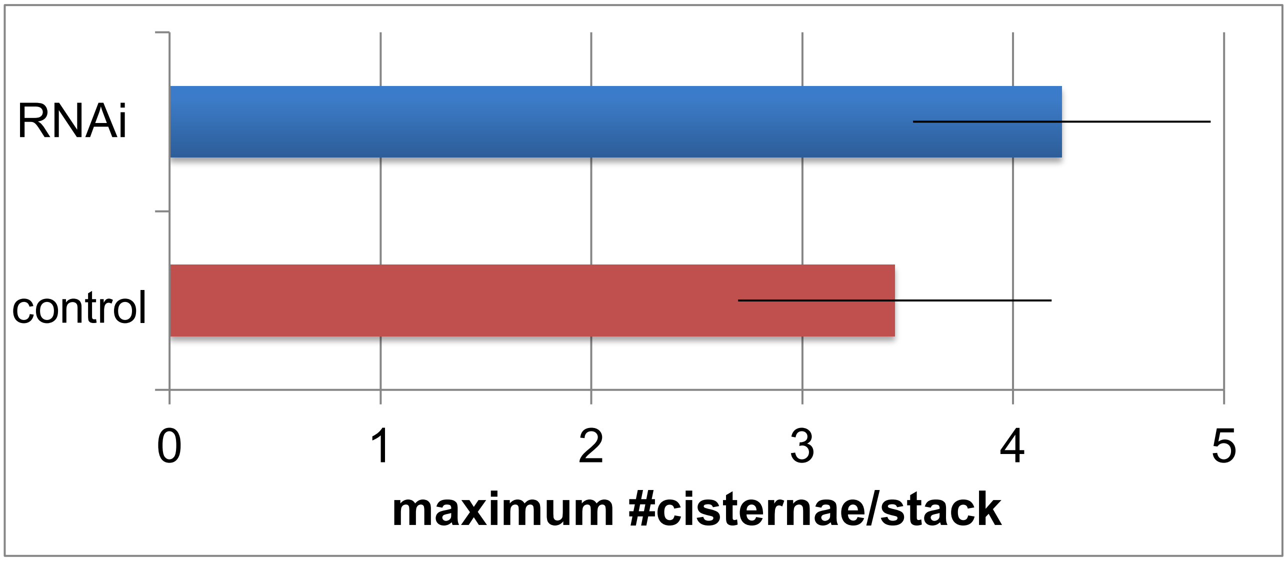

Supplementary Figure 3. Loss of Giantin elongates Golgi cisternae. The numbers of cisternae per Golgi stack were counted manually and the mean numbers are shown (n=40, bar, SD).

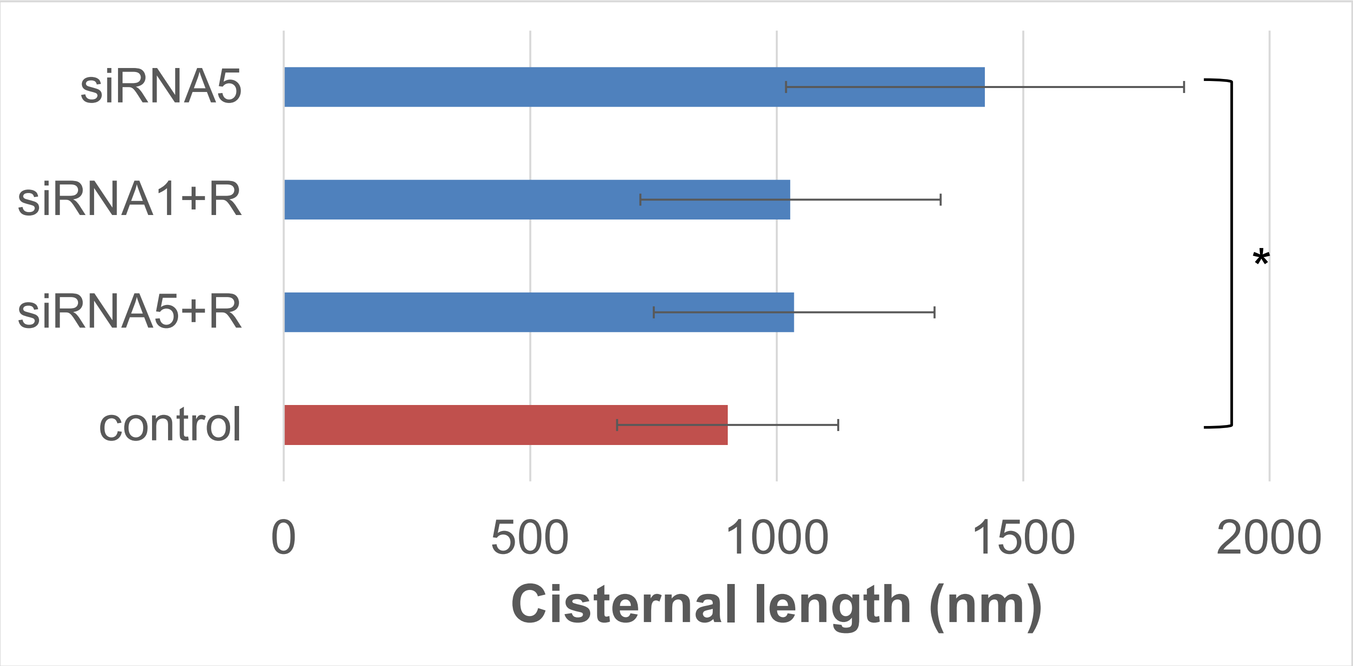

Supplementary Figure 4. Loss of Giantin elongates Golgi cisternae.

Another siRNA (siRNA5) showed the similar effect in cisternal length. Exogenous expression of Giantin (+R) restored cisternal length partly. Cisternal lengths were measured by tracing the cisternae manually using Image J software. (n=~60 micrographs, bar, SD, **P*<0.00001 was calculated using an online calculator (https://www.socscistatistics.com/tests/mannwhitney/).).

4 Additional Control Golgi Stacks

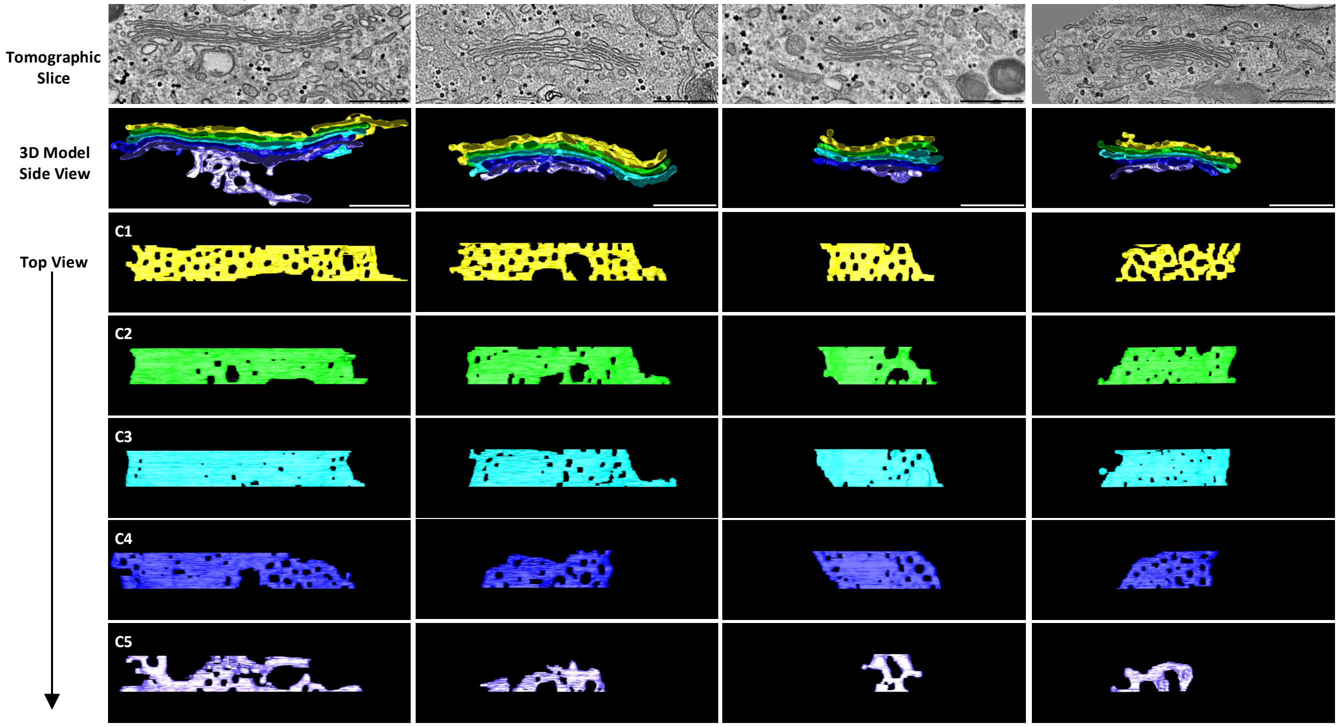

4 Additional RNAi Golgi Stacks

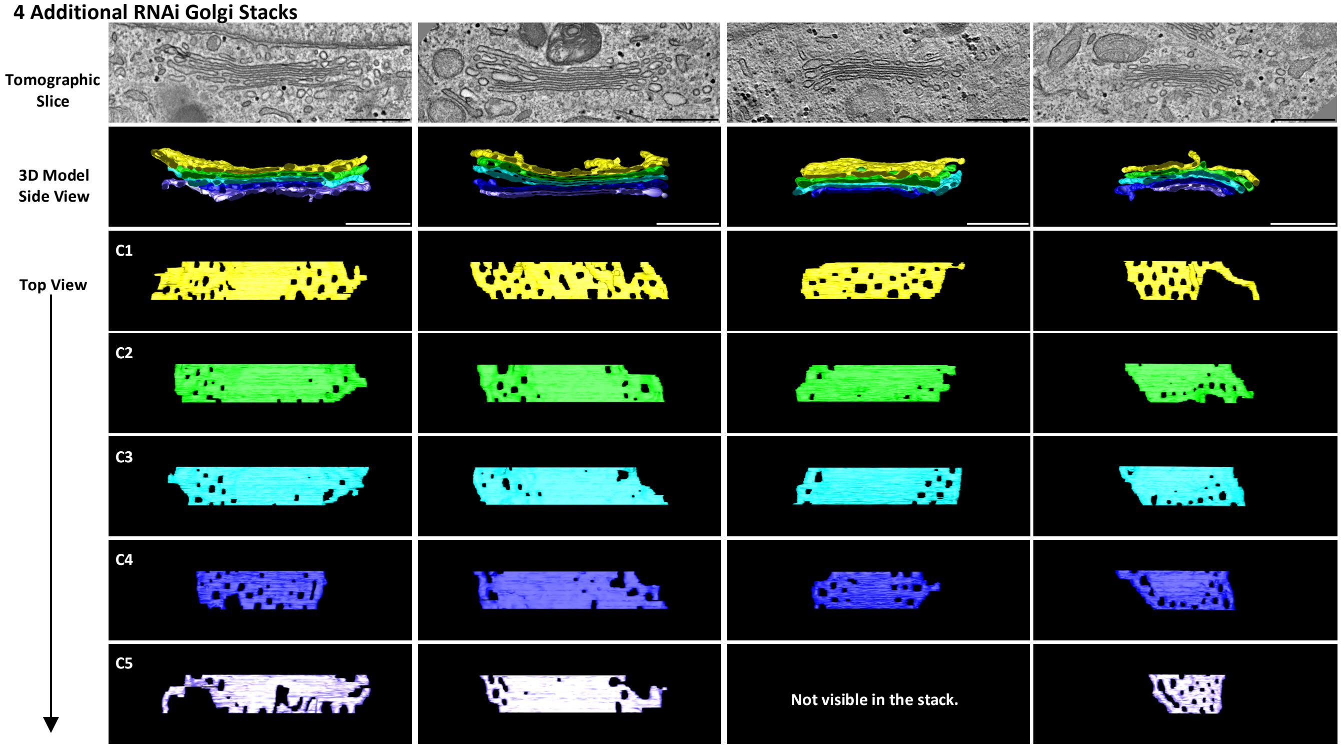

Supplementary Figure 5. Loss of Giantin fuses Golgi cisternae.

Additional 3D models of Golgi cisternae in Giantin and control siRNA-treated cells. One of the tomographic slices used for these 3D models is shown in top panels. Note that the Golgi cisternae visible in RNAi cells appear longer than those in control cells. Bar, 500 nm. The cis-most and trans-most cisternae are labeled C1 and C5, respectively. Corresponding tomograms (movies) are available as Supplementary movies.

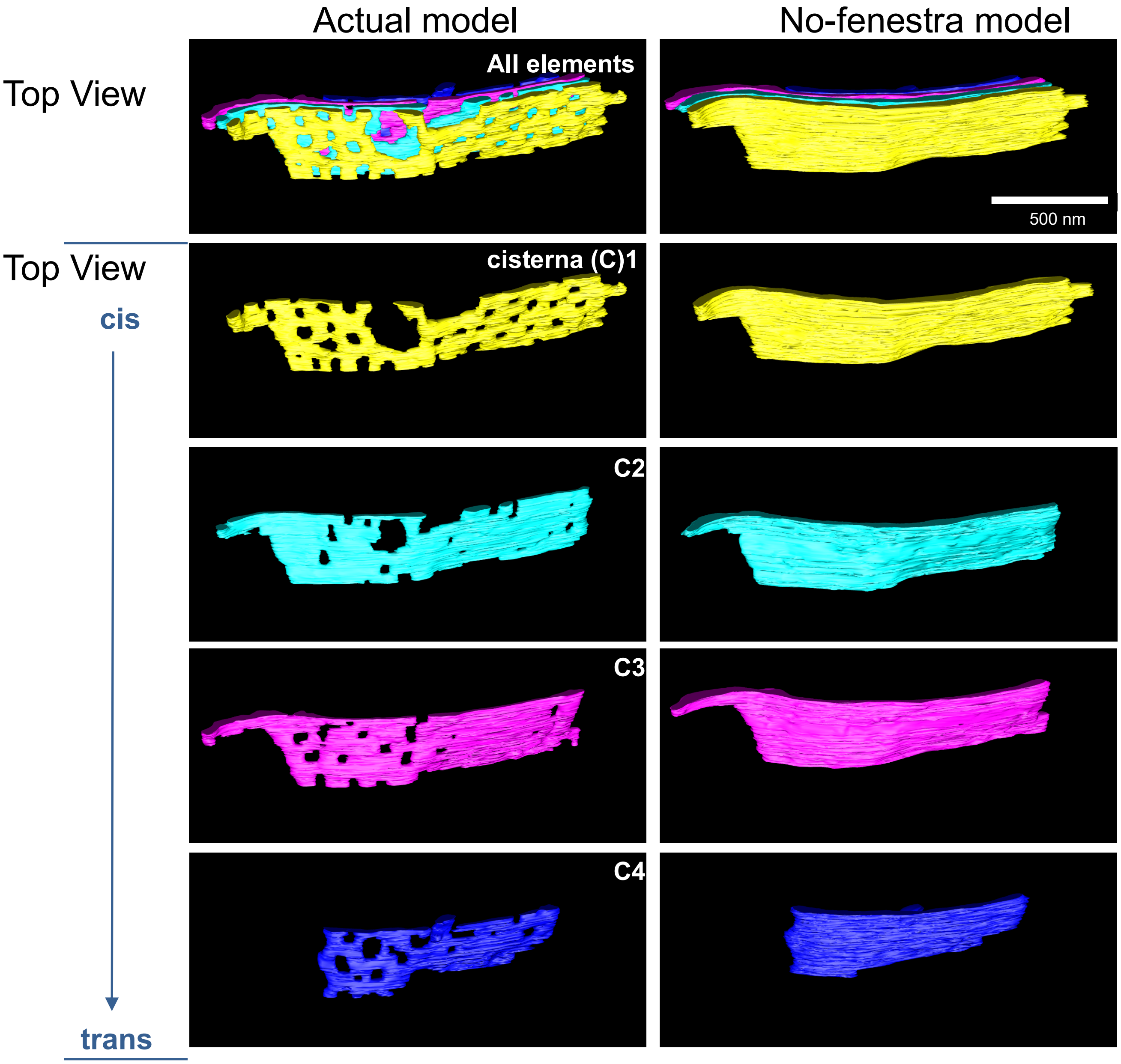

Supplementary Figure 6. An example of a no-fenestra model.

Typical 3D models of "actual" and "no-fenestra" from control cells. Bar, 500 nm.

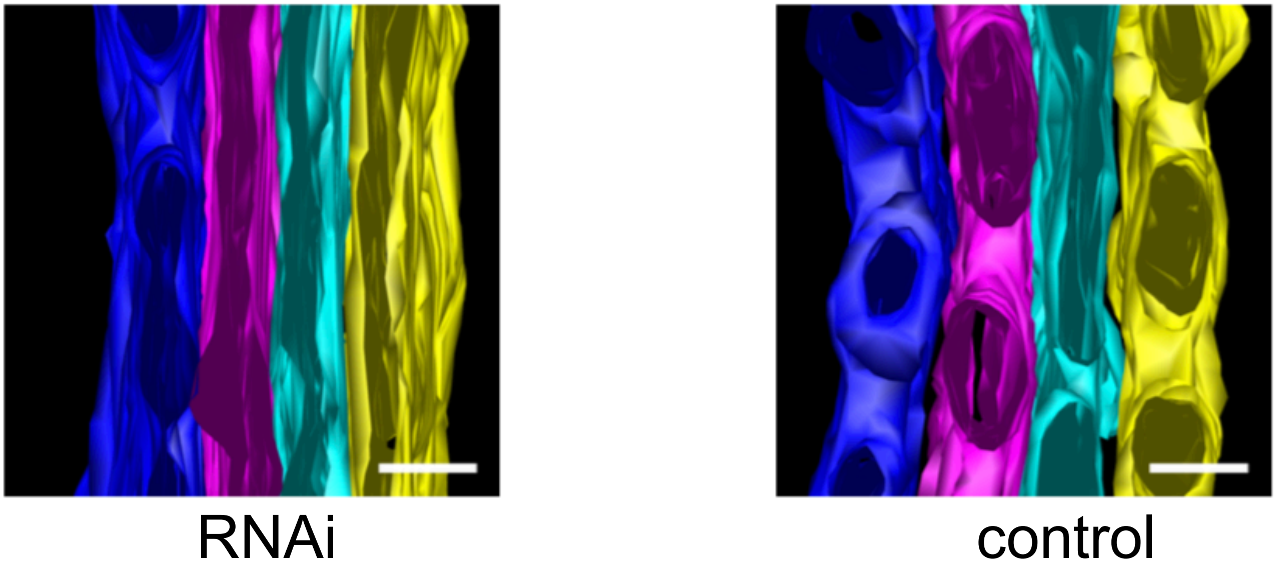

Supplementary Figure 7.

Enlarged 3D models to show thicker cisternae around the fenestrae. Bar, 50 nm.

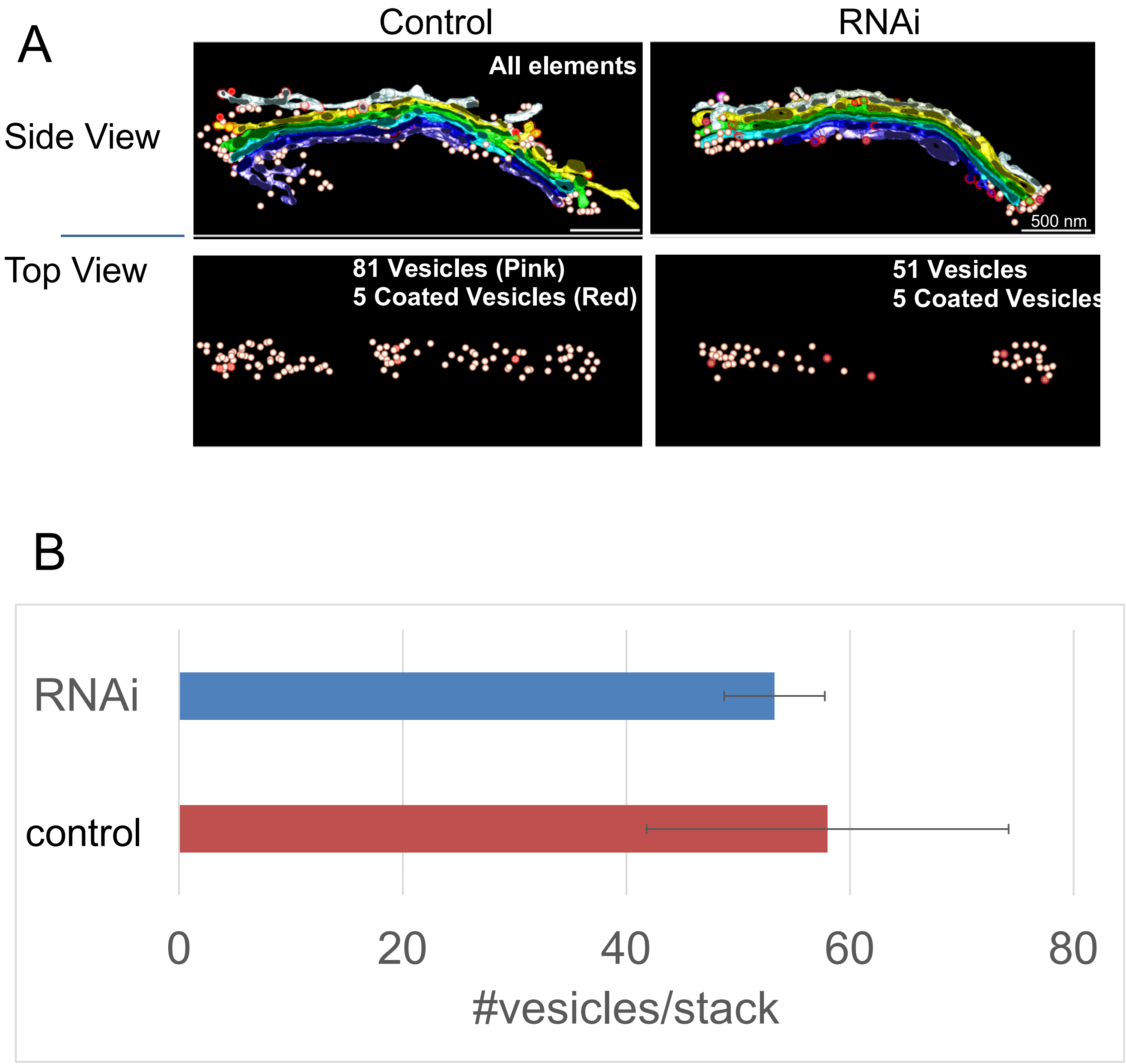

Supplementary Figure 8. Loss of Giantin does not affect numbers of vesicles around Golgi cisternae. (A) Typical 3D models of Golgi cisternae in Giantin and control siRNA-treated cells shown in Figure 1A presented with coated and uncoated vesicles in red and pink, respectively. Bar, 500 nm. (B) Vesicles were modeled and counted, and shown as the #vesicles/tomogram. Bar, SD (n=4, **P*>0.6).

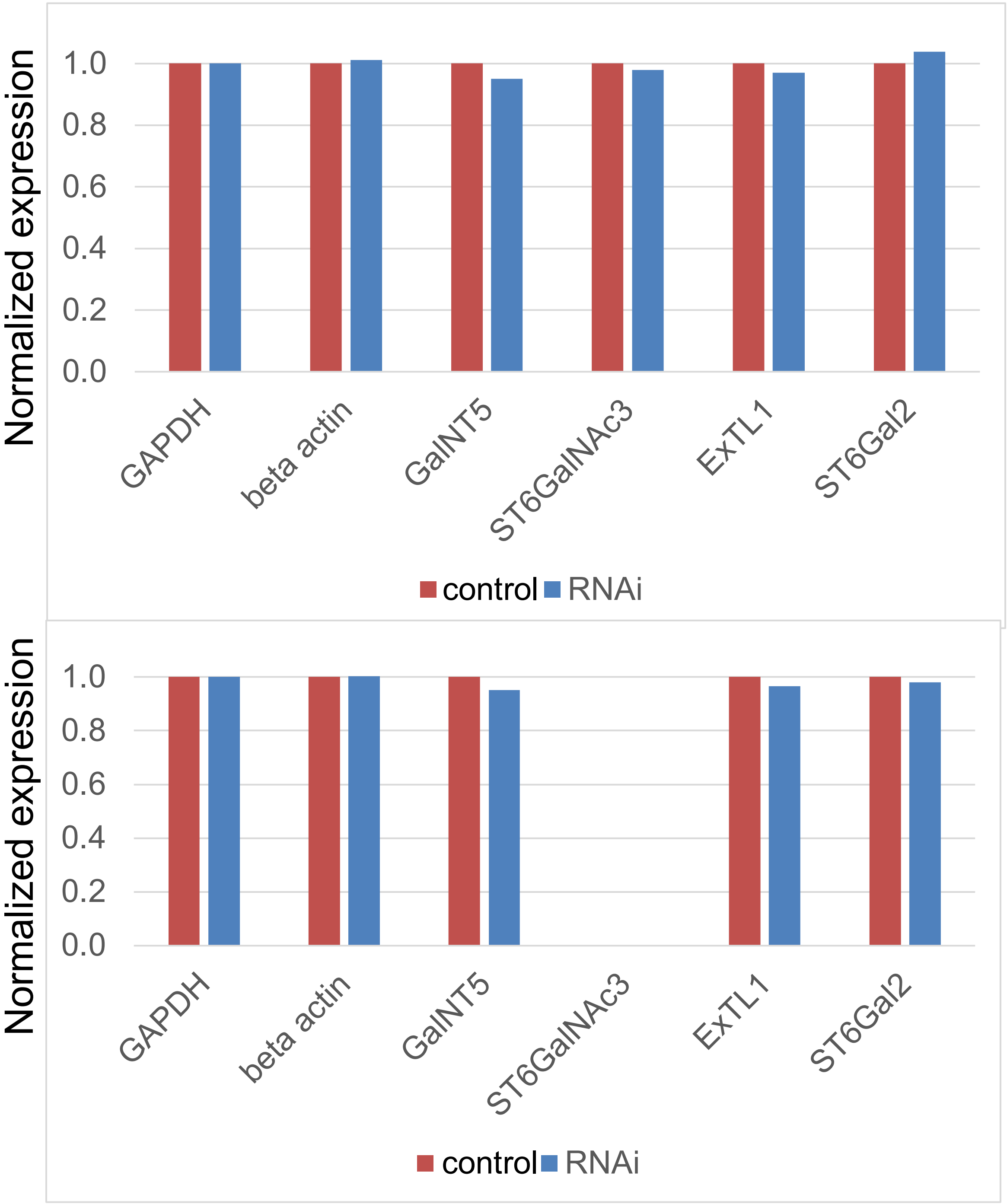

Supplementary Figure 9. Relative expression of glycosyltransferases in HeLa with or without Giantin KD.

Total RNA of Giantin and control siRNA-treated cells were isolated and subjected for qPCR. The relative mRNA expression (normalized with those from control cells) of indicated genes were shown in upper and lower graphs of 2 independent experiments.

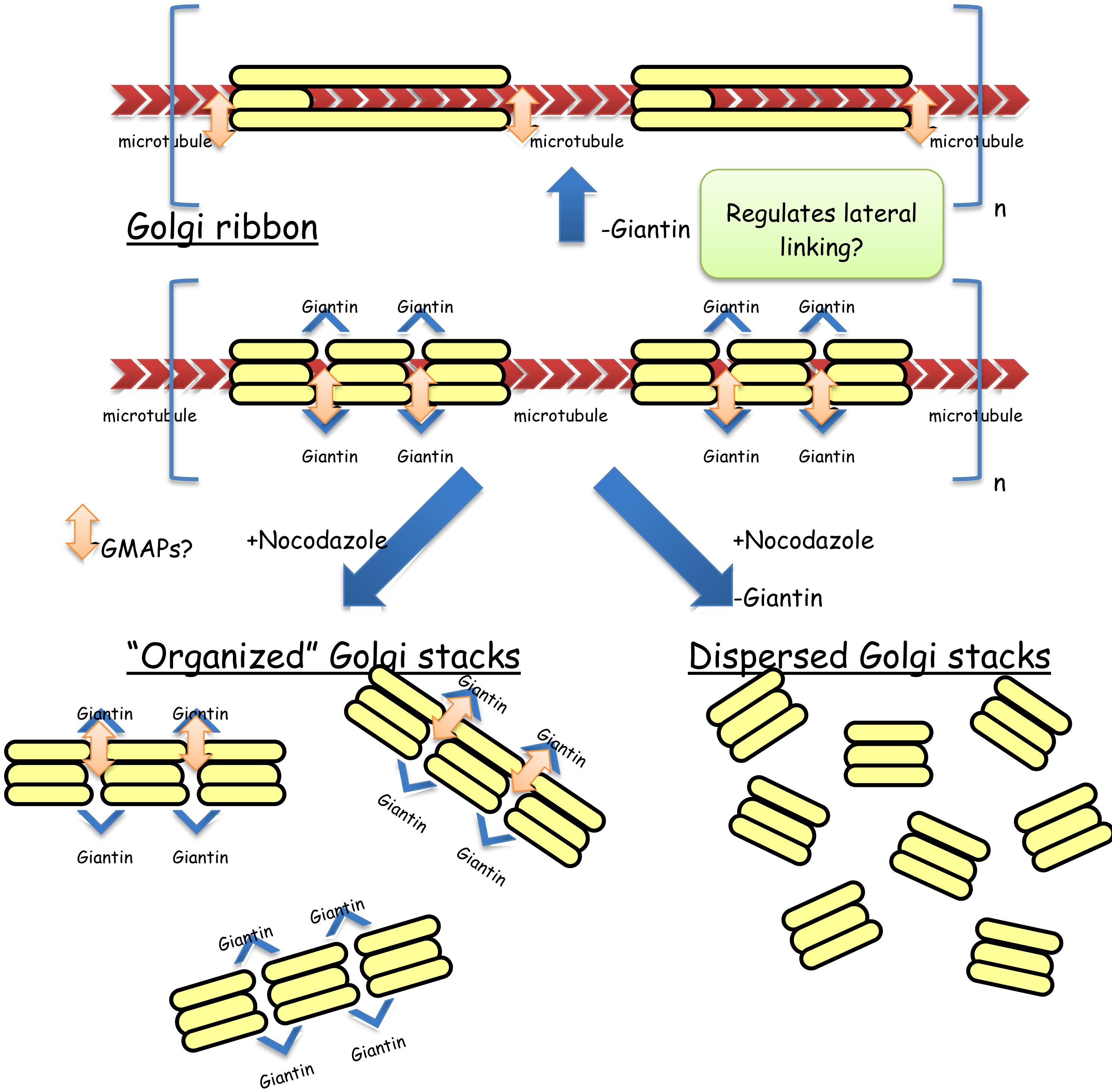

Supplementary Figure 10. Updated working model.

The present work showed that the loss-of-Giantin connects Golgi cisternae in HeLa cells (top diagram). This may be consistent with our previous model by adding an unknown molecule which links microtubules, Giantin and/or Golgi (lower diagrams). The candidate for such molecules may be GMAPs. This diagram was drawn with modifications of our previous model shown in https://doi.org/10.1371/journal.pone.0059821.s002.

Supplementary Table 1

	Control	siRNA1	siRNA5	siRNA1+r*	siRNA5+r*
%Max. recovery**	21.71±0.49	40.00±0.57	47.69±0.23	35.95±0.24	30.15±0.88
Relative diffusion**	26.83±0.60	60.98±0.87	94.66±0.41	37.33±0.25	36.59±1.07

*rescue construct transfected.

**calculated using Ellenberg's diffusion equation [ref 54].

Supplementary movies

Video 1. Tomograms of Figure 3AB (control)
Video 2. Tomograms of Figure 3AB (RNAi)
Video 3. Additional tomograms of control 1
Video 4. Additional tomograms of control 2
Video 5. Additional tomograms of control 3
Video 6. Additional tomograms of control 4
Video 7. Additional tomograms of RNAi 1
Video 8. Additional tomograms of RNAi 2
Video 9. Additional tomograms of RNAi 3
Video 10. Additional tomograms of RNAi 4
Video 11. Tomograms of another siRNA to Giantin (another siRNA, labeled as siRNA5) 1
Video 12. Tomograms of another siRNA to Giantin (another siRNA, labeled as siRNA5) 1
Video 13. Tomograms of another siRNA to Giantin (another siRNA, labeled as siRNA5) 1
Video 14. Tomograms of another siRNA to Giantin (another siRNA, labeled as siRNA5) 1
Video 15. Tomograms of another siRNA to Giantin (another siRNA, labeled as siRNA5) 1
